# The N-terminal domain of a tick evasin is critical for chemokine binding and neutralization and confers specific binding activity to other evasins

**DOI:** 10.1074/jbc.RA117.000487

**Published:** 2018-02-27

**Authors:** James R. O. Eaton, Yara Alenazi, Kamayani Singh, Graham Davies, Lucia Geis-Asteggiante, Benedikt Kessler, Carol V. Robinson, Akane Kawamura, Shoumo Bhattacharya

**Affiliations:** From the ‡ Division of Cardiovascular Medicine, Radcliffe Department of Medicine,; the § Department of Chemistry, and; the ¶ Nuffield Department of Medicine, University of Oxford, Oxford OX3 7BN, United Kingdom

**Keywords:** chemokine, chemotaxis, protein–protein interaction, inflammation, mass spectrometry (MS), homology modeling, anti-inflammatory therapy, domain swapping, evasin, tick

## Abstract

Tick chemokine-binding proteins (evasins) are an emerging class of biologicals that target multiple chemokines and show anti-inflammatory activities in preclinical disease models. Using yeast surface display, we identified a CCL8-binding evasin, P672, from the tick *Rhipicephalus pulchellus*. We found that P672 binds CCL8 and eight other CC-class chemokines with a *K_d_* < 10 nm and four other CC chemokines with a *K_d_* between 10 and 100 nm and neutralizes CCL3, CCL3L1, and CCL8 with an IC_50_ < 10 nm. The CC chemokine–binding profile was distinct from that of evasin 1 (EVA1), which does not bind CCL8. We also show that P672's binding activity can be markedly modulated by the location of a StrepII-His purification tag. Combining native MS and bottom-up proteomics, we further demonstrated that P672 is glycosylated and forms a 1:1 complex with CCL8, disrupting CCL8 homodimerization. Homology modeling of P672 using the crystal structure of the EVA1 and CCL3 complex as template suggested that 44 N-terminal residues of P672 form most of the contacts with CCL8. Replacing the 29 N-terminal residues of EVA1 with the 44 N-terminal residues of P672 enabled this hybrid evasin to bind and neutralize CCL8, indicating that the CCL8-binding properties of P672 reside, in part, in its N-terminal residues. This study shows that the function of certain tick evasins can be manipulated simply by adding a tag. We conclude that homology modeling helps identify regions with transportable chemokine-binding functions within evasins, which can be used to construct hybrid evasins with altered properties.

## Introduction

Chemokines are a family of secreted proteins that are major drivers of both physiological and pathological inflammation (1). They fall into four groups, CC, CXC, XC, and CX3C, which are defined by the spacing between their N-terminal cysteine residues (2). Structural features common to all chemokines include: a flexible N terminus followed by the double cysteine motif, the N-loop, a helix, a three-stranded sheet with 30S (between β1 and β2) and 40S (between β2 and β3) loops, and a C-terminal helix. The distal N terminus of the chemokine binds to the chemokine recognition site 2 (CRS2)^6^ pocket of G-protein–coupled chemokine receptors, whereas the proximal N terminus and N-loop/40S loop grooves bind chemokine recognition site 1 (CRS1) in the extracellular N terminus of the receptor (3, 4). Binding to the CRS2 pocket results in conformational changes in the G-protein–coupled receptor that activates signaling. Binding of chemokines to target receptors is typically a “one-to-many” interaction, with several chemokines binding more than one receptor and several receptors binding more than one chemokine. Chemokines also bind with low affinity to endothelial cell surface glycosaminoglycans, which is necessary for chemokine function *in vivo* (5). The 19 chemokine receptors that activate chemotaxis are expressed in varying combinations on adaptive and innate immune cells, and activation by chemokines promotes their directional migration to sites of chemokine expression (6–8). The one-to-many interactions between chemokines and receptors, the expression of multiple receptor types on immune and inflammatory cells, and the expression of multiple chemokines at the site of injury or diseased tissues leads to the creation of a robust network that is not amenable to traditional therapeutics that monovalently target either chemokines or their receptors.

Natural selection in viruses, helminths, and ticks has resulted in the convergent evolution of proteins that target the chemokine network by different mechanisms (reviewed in Refs. 9 and 10). For instance, the viral proteins MC148 and vMIP-II bind and inhibit multiple chemokine receptors. Other viruses have evolved chemokine-binding proteins (CKBPs) that bind multiple CC and CXC chemokines and disrupt glycosaminoglycan binding or disrupt chemokine binding to the receptor or both (11). Helminth CKBP also binds multiple CC and CXC chemokines, but the mechanism of action is not clearly understood (12). Ticks, uniquely, have two classes of CKBPs, called evasins, with one class typically containing eight conserved Cys residues (“8-Cys” tick CKBPs) that bind subsets of CC chemokines and another class containing six conserved Cys residues (“6-Cys” CKBPs) that bind CXC chemokines. Following the initial identification of class I tick CKBPs, exemplified by evasin 1 (EVA1_RHISA, abbreviated to EVA1) and evasin 4 (EVA4_RHISA, abbreviated to EVA4), and a class II CKBP, exemplified by evasin 3 (EVA3_RHISA (13), abbreviated to EVA3), we (14) and others (15) have identified several other examples of CC-CKBPs from diverse tick species. The structure of EVA1 in complex with CCL3 shows that it has 1:1 stoichiometry, with the N and C termini of EVA1 binding the N terminus and N-loop of CCL3 (16). The selectivity of the EVA1–CCL3 interaction resides in the six residues that immediately precede the double cysteine motif in CCL3. Binding results in a conformational change of the N terminus of CCL3, and this, together with the binding of the N-loop, explains the inhibition of chemokine activity (16). *In silico* modeling and mutagenesis studies indicate that EVA4 also targets the CCL3 N terminus (17). Both EVA1 and EVA4 have been shown to have anti-inflammatory efficacy in several preclinical disease models, suggesting that they may be potential therapeutics in inflammatory disease (13).

Using yeast surface display, we have isolated a novel CCL8-binding CKBP from the tick *Rhipicephalus pulchellus* called P672_RHIPU (abbreviated to P672). We show that P672 binds and neutralizes several CC chemokines and that its ability to bind and neutralize chemokines is modulated by the location of a purification tag. We show that binding to CCL8 occurs with 1:1 stoichiometry and disrupts CCL8 homodimer formation. Using molecular modeling we identified a CCL8-binding region in P672 that can be transplanted to EVA1, a protein that does not bind CCL8. These results show that tick CKBP function can be modulated in some cases by the addition of a tag, providing proof of the concept that regions within tick CC-CKBPs with transportable properties can be identified and manipulated to create non-natural hybrid proteins with altered characteristics.

## Results

### Screening of a yeast surface display library

**Figure 1.**
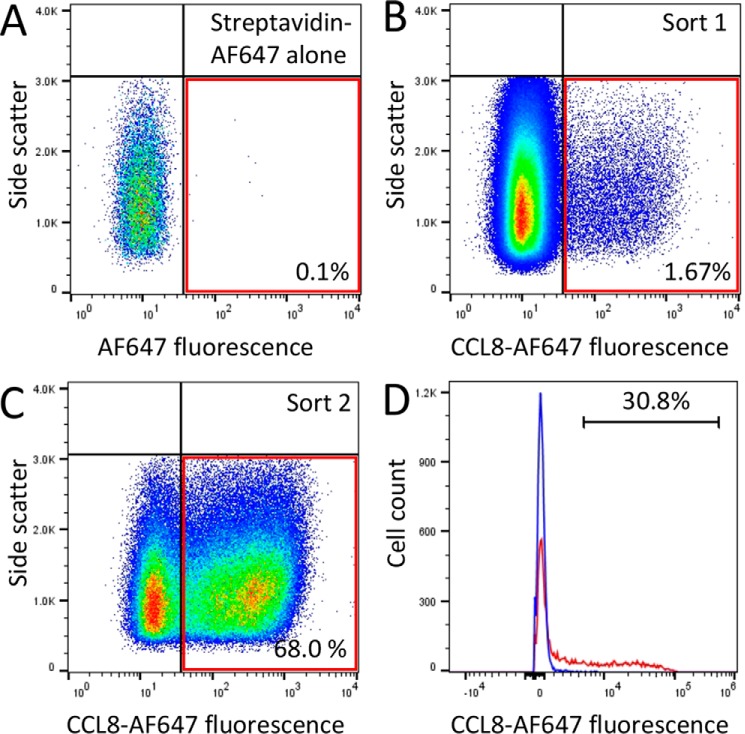
**Yeast surface display screen.** *A*, fluorescence profile of yeast surface display library expressing putative tick evasins incubated with streptavidin-AF647 alone. The *red outlined box* indicates the sorting gate. *B*, yeast surface display library incubated with biotinylated CCL8 plus streptavidin-AF647. The sorting gate was identified from the negative control and was used to sort CCL8-binding yeast from the library. *C*, second round sorting of CCL8-binding yeast identified above. The proportions of cells within the sorting gate are indicated as a percentage. *D*, binding of P672 to CCL8. Yeast expressing P672 are shown in *red*, and control yeast bearing empty vector surface display plasmid are shown in *blue*. The *y* axis shows cell count and *x* axis the fluorescence intensity on a log_10_ scale. The proportion of cells exceeding background is indicated as a percentage.

We used fluorescence-activated cell sorting (FACS) to screen a yeast surface display library as described in detail previously (14). The plasmid library was constructed to express 352 putative tick CKBPs with either N-terminal (AGA2) or C-terminal (SAG1 or AGA2) surface display tags. The plasmid library was transformed as a pool into yeast, and the cells were labeled with biotinylated CCL8 and streptavidin-AF647. The labeled cells were sorted by two rounds of FACS (Fig. 1, *A* and *B*). In each round, we used a sorting gate determined by FACS analysis of the yeast cells with streptavidin-AF647 alone. The sorting gate was used to exclude yeast cells displaying background fluorescence generated by streptavidin-AF647 binding to yeast. Cells that were recovered in the first sort were regrown and then sorted once again (Fig. 1*C*). Cells recovered in the second sort were next plated at low density, and individual yeast clones were picked and retested by labeling them with biotinylated CCL8 and streptavidin–Alexa Fluor 647. The clones picked were compared with control yeast expressing the relevant surface display tag (Fig. 1*D*). In these experiments, we found that only a proportion (30.8%) of the clonal yeast population would bind CCL8. Two independent clones recovered in this screen encoded a novel tick CKBP, P672 (GenBank^TM^ accession number JAA60789.1). Both clones were obtained with an N-terminal surface display tag.

### Sequence analysis of P672

**Figure 2.**
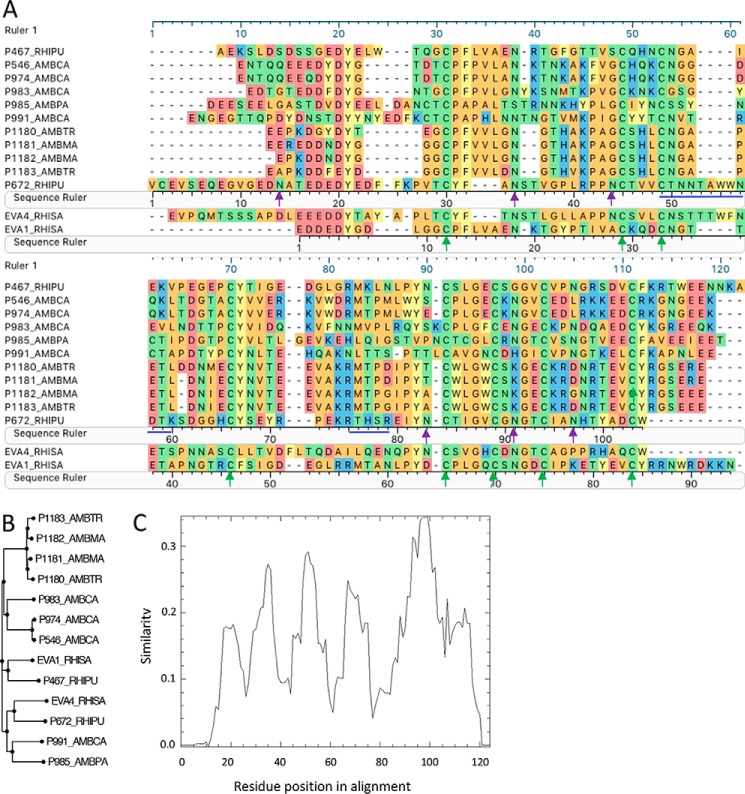
**Analysis of P672 protein sequence.** *A*, sequence alignment of P672 with CC-binding evasins. The peptide sequence prefix indicates the identity, and suffixes indicate the tick species as follows: RHISA and RHIPU, *Rhipicephalus sanguineus* and *R. pulchellus*, respectively; AMBPA, AMBCA, AMBMA, and AMBTR, *Amblyomma parvum*, *Amblyomma cajennense*, *Amblyomma maculatum*, and *Amblyomma triste*, respectively. Amino acid residues are color-coded according to physicochemical properties: *yellow*, aromatic (*F*, *W*, and *Y*); *red*, acidic (*D* and *E*); *blue*, basic (*R*, *H*, and *K*); *orange*, nonpolar aliphatic (*A*, *G*, *I*, *L*, *M*, *P*, and *V*); *green*, polar neutral (*C*, *N*, *Q*, and *T*). The *green arrows* indicate disulfide bond positions in EVA1. *Magenta arrows* indicate the six *N*-glycosylated residues identified in P672 by mass spectrometry. The *blue lines* indicate the regions of P672 not covered by elastase and tryptic digest bottom-up mass spectrometry. Sequence rulers of P672, EVA1, and the alignment (ruler 1) are indicated. *B*, phylogenetic tree of CC-binding evasins. P672 is related most closely to EVA4, with which it shares 36% identity over 105 residues. *C*, conservation plot of evasins identified by yeast surface display. The plot was generated from the sequence alignment using the EMBOSS program Plotcon with a window size of 10 residues. The *y* axis shows the similarity score, and the *x* axis shows the residue position in the alignment.

P672 has 104 amino acid residues with a predicted molecular mass of 11.7 kDa and a predicted isoelectric point (pI) of 4.45. The glycosylation site prediction indicates several *N*-glycosylations and a single *O*-glycosylation site. Protein sequence alignment with EVA1, EVA4, and other CC-CKBPs identified by yeast surface display shows that P672 retains the eight Cys residues that form disulfide bonds in EVA1. The arrangement of the Cys residues is consistent with the motif C*X*(14,17)C*X*(3)C*X*(11,16)C*X*(17,20)C*X*(4)C*X*(4)C*X*(8)C that we have described previously (14) (Fig. 2*A*). Phylogenetic analysis of the protein sequence shows that P672 is most closely related to EVA4 (Fig. 2*B*), with which it shares 36% identity over an alignment length of 105 residues. It shares only 29% identity with EVA1, over an alignment length of 89 residues. There is no significant homology to EVA3. The Pro residue in EVA1 that immediately follows the first Cys residue and contacts the disulfide bond in CCL3 (18) is replaced by a Tyr residue, as is the case for EVA4. The conservation plot (Fig. 2*C*) shows that the N and C termini of CC-CKBPs are less well conserved than the central core region.

### Expression and purification of P672

**Figure 3.**
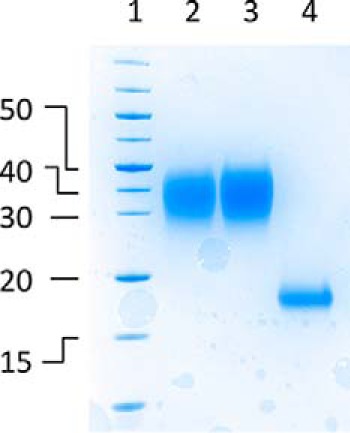
**Analysis of glycosylated and deglycosylated P672 by SDS-PAGE.** The gel is stained with colloidal Coomassie Blue. Shown are molecular mass markers (kDa, *lane 1*), N-terminally tagged P672 protein (*lane 2*), C-terminally tagged P672 protein (*lane 3*), and N-terminally tagged P672 protein (*lane 4*), partially deglycosylated using PNGaseF and EndoF1. All proteins were purified using nickel affinity followed by size exclusion chromatography.

We expressed P672 as a secreted protein from mammalian cells essentially as described previously (14). We purified P672 with a C-terminal StrepII-His purification tag by nickel affinity followed by size exclusion chromatography. However, as P672 was obtained only in an N-terminally tagged orientation in the yeast surface display screen, we also used an N-terminal His-StrepII tag approach to purify the protein. The purified protein in each case migrated at a larger molecular mass than expected (Fig. 3 and Fig. S1). This is consistent with its predicted glycosylation, which was confirmed by digestion of the protein with the glycosidases PNGaseF and Endo F1. On digestion, the smeared band collapses to a single band close to the predicted molecular mass on SDS-PAGE (Fig. 3) together with a corresponding change in retention volume on size exclusion chromatography (Fig. S1*A*).

### Tandem mass spectrometry identification of glycosylated sites in P672

Tandem mass spectrometry (MS/MS) analysis at the peptide level of P672 treated with PNGaseF and Endo F1 was performed, and we were able to achieve sequence coverage of 87%, excepting the residues indicated in Fig. 2*A*. This identified several deamidated residues that had converted from Asn to Asp, corresponding to a mass shift of +0.98 Da in N*X*(S/T) motifs (supplemental material Table S1). Deamidation of Asn is a characteristic feature of *N*-glycosylation cleavage by PNGaseF (19) and indicates that the Asn residues are *N*-glycosylated. Only partial deglycosylation by PNGaseF and Endo F1 was achieved as supported by the finding of several peptides in deamidated and glycosylated forms carrying *N*-acetylglucosamine and/or *N*-acetylgalactosamine (HexNAc) moieties. The presence of HexNAc can be explained by the Endo F1 cleavage. The *N*-glycosylation sites identified by MS/MS are indicated in Fig. 2*A*.

### Binding of P672 to chemokines

**Figure 4.**
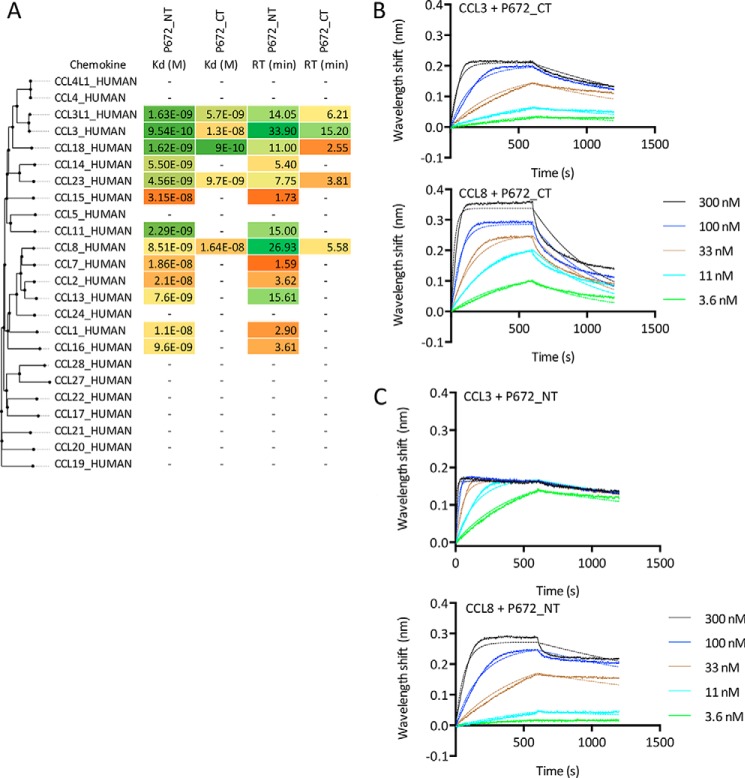
**Characterization of P672 binding to chemokines using biolayer interferometry.** *A*, binding affinities (*K_d_*, m) and target residence times (*RT*, minutes) of immobilized P672 (either N- or C-terminally tagged as indicated) to human CC-chemokines using biolayer interferometry. High-affinity binding and longer residence times are indicated as shades of *green*, medium affinity as *yellow*, and low affinity as shades of *orange*. Chemokines are arranged by sequence similarity–based phylogeny. A *dash* (—) indicates that binding was not detected at 300 nm chemokine concentration. *B*, biolayer interferometry sensorgrams showing C-terminally tagged P672 binding to different doses of chemokines CCL3 (*top panel*) and CCL8 (*bottom panel*). Plots display wavelength shift (*y* axis, nm) *versus* time (*x* axis, seconds). *Solid lines* indicate collected data, and *dashed lines* indicate fitted data. *C*, biolayer interferometry sensorgrams showing N-terminally tagged P672 binding to different doses of chemokines CCL3 (*top panel*) and CCL8 (*bottom panel*). Plots display wavelength shift (*y* axis, nm) *versus* time (*x* axis, seconds). *Solid lines* indicate collected data, and *dashed lines* indicate fitted data.

We next explored the binding profile of glycosylated P672, with both the N- and C-terminally tagged forms to a chemokine panel using biolayer interferometry (20). CKBPs were immobilized on the sensor through their His tag, and initial screening assays performed at 300 nm chemokine concentration indicated that P672 bound only certain CC chemokines. No binding was detected to CXC, XC, or CX3C chemokines (data not shown). Binding affinities (*K_d_*) were subsequently determined through biolayer interferometry titration studies against these CC chemokines (Fig. 4). Notably, we observed different binding patterns when P672 was tagged at the C terminus, specifically the loss of binding to CCL14, CCL15, CCL11, CCL7, CCL2, CCL13, CCL1, and CCL16 at 300 nm chemokine concentration. We also noted a marked variation in target residence time (calculated from the off-rate) with P672 tag positioning, *e.g.* for its binding to CCL8.

### Neutralization of chemokines by P672

**Figure 5.**
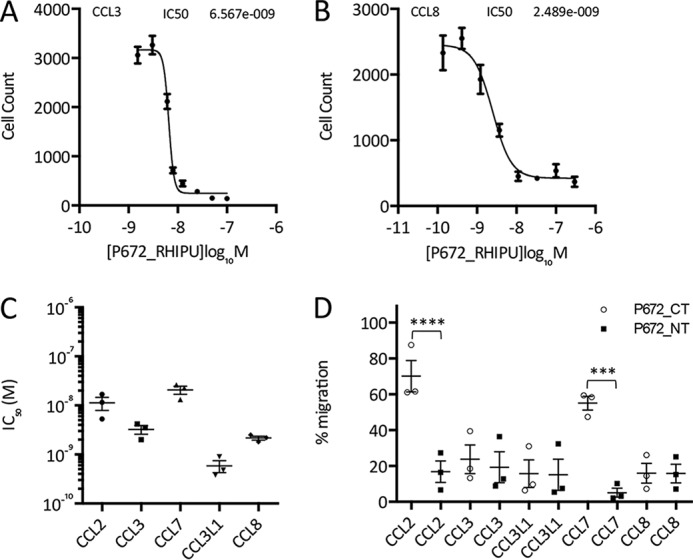
**Functional neutralization of chemokine activity by P672.** *A* and *B*, neutralization of CCL3 (*A*)- and CCL8 (*B*)-induced THP-1 cell migration by N-terminally tagged P672. The *y* axis shows cell count of THP-1 cells migrating through to the bottom chamber in response to an EC_80_ dose of the indicated chemokines. Data (three technical replicates) are shown as mean ± S.E. The *x* axis shows P672 concentration (log_10_ molar). The IC_50_ indicated was estimated by fitting an agonist response curve with four parameters as described (14). *C*, summary of N-terminally tagged P672 IC_50_ data (*M*, mean ± S.E. of three biological replicates analyzed as above) for chemokines that induce THP-1 cell migration. The EC_80_ doses of the indicated chemokines were as follows: CCL2, 0.52 nm; CCL3, 3.55 nm; CCL7, 7.18 nm; CCL3L1, 0.52 nm; and CCL8, 5.75 nm. *D*, percentage of migration inhibition by a fixed concentration (100 nm) of P672_CT (C-terminally tagged (*open circles*)) or P672_NT (N-terminally tagged (*filled squares*)). The experiment was performed at an EC_80_ concentration of the chemokines indicated. Data (three technical and three biological replicates) were normalized to chemokine-alone control to account for day to day variation. Data were analyzed with one-way analysis of variance and corrected for multiple comparisons with the Sidak method. ***, *p* < 0.001; ****, *p* < 0.0001.

We next explored the ability of P672, in N- and C-terminally tagged versions, to inhibit chemokine function. We monitored their effects on the migration of THP-1 human monocyte cells in response to human CC chemokines in 96-well Boyden chamber assays (Fig. 5). We determined the effect of titrating in progressively increasing doses of P672 at the effective concentration (EC) dose, EC_80_, for each CC chemokine and found that N-terminally tagged P672 inhibits THP-1 cell migration in response to CC chemokines with IC_50_ <10 nm (Fig. 5, *A–C*). The relative effects of tag position were determined by comparing the ability of the N- or C-terminally tagged form of P672 to inhibit THP-1 cell migration (Fig. 5*D*). The C-terminal tag position had marked effects on the ability of P672 to inhibit CCL2- and CCL7-activated chemotaxis. Tag position had no significant effect on the ability of P672 to inhibit CCL3, CCL3L1, or CCL8 function. This is consistent with the binding data presented above and indicates that tag position can affect not only the binding of P672 to chemokines but also its function in solution.

### Characterization of P672 in complex with CCL8

To understand the mechanism of P672 binding to CCL8, we characterized individual proteins and the complex using native mass spectrometry. As glycosylation can affect native mass spectrometry unpredictably, we used partially deglycosylated P672 for these experiments. The affinity of partially deglycosylated N-terminally tagged P672 for CCL8 was 7.25E-8 m (Fig. S1*B*), indicating that native mass spectrometry of the P672-CCL8 complex would be feasible and would allow us to elucidate the stoichiometry of the interaction.

**Figure 6.**
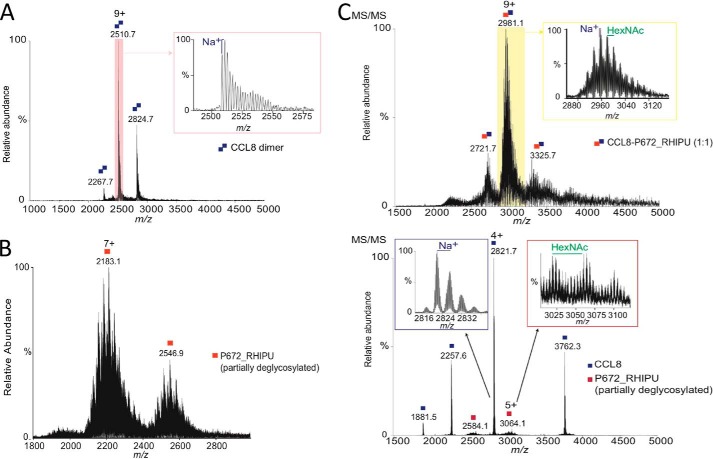
**Analysis of the CCL8-P672 complex using native mass spectrometry.** *A*, native MS of CCL8 homodimer. *Inset* shows sodium adducts of the homodimer. *B*, native MS of partially deglycosylated N-terminally tagged P672. *C*, *top panel* and *inset*, native MS of P672-CCL8 complex. CCL8 and P672 were mixed at a 1:1 ratio (final concentration of 2.5 μm) and incubated for 1 h at 4 °C. Peaks corresponding with a glycosylated and sodiated CCL8-P672 heterodimer (lowest intensity average mass observed is 25,915.3 ± 1.4 Da) are indicated (*inset*). *Bottom panel*, HCD fragmentation at 100 V of the *m*/*z* 2956 isolated using a 100 *m*/*z* isolation window. Peaks corresponding to sodiated CCL8 (*top left inset*) and a partially glycosylated P672 (*top right inset*) are indicated. In all sections of this figure, the peaks are indicated as CCL8 (*blue squares*) or P672 (*red squares*). Monomers are indicated as a *single square* and homo- or heterodimers as *double squares*. The *y* axis indicates relative abundance, and the *x* axis indicates the *m*/*z* (mass/charge) ratio.

Initially, CCL8 and P672 were characterized separately. CCL8 was expressed bacterially as an N-terminal His-SUMO fusion, which following SUMO cleavage produced a new N terminus corresponding to glutamine (Gln-24). The presence of the glutamine N-terminal residue was confirmed using high resolution native MS/MS. The N-terminal glutamine of CCL8 can cyclize to form pyroglutamic acid, which is required for CCL8 biological activity (21). During electrospray ionization the N-terminal glutamine can also readily cyclize to form pyroglutamic acid. This change corresponded to the most intense peak observed, and the identity and site of modification were supported by higher-energy collision dissociation (HCD) fragment ions (Fig. S2). Purified CCL8 was biologically active in a cell migration assay (data not shown). We found that CCL8 formed a homodimer under native mass spectrometric conditions with an average mass of 22,566.6 ± 0.8 Da (Fig. 6*A*). In the case of P672 treated with PNGaseF and Endo F1, native MS spectra were more complex due to the presence of partial glycosylations, deamidations, and sodium adducts (Fig. 6*B*). The average masses observed ranged from 14,588 to 16,628 Da depending on the modifications present. Moreover, mass differences of ∼203 Da were observed, suggesting the presence of HexNAc moieties, which supports the results obtained by bottom-up proteomics (Fig. S3).

Native mass spectrometry of the CCL8-P672 complex (mixed in at a 1:1 ratio) showed peaks of average masses ranging from 25.9 to 27.9 kDa, which corresponds well with that expected for a glycosylated and sodiated CCL8-P672 heterodimer (Fig. 6*C*, *top panel*). To demonstrate that this was the CCL8-P672 complex, the *m*/*z* 2956 was isolated with a 100 *m*/*z* isolation window and fragmented with HCD at 100 V. We observed that the complex clearly dissociated into CCL8 and a partially glycosylated P672 (Fig. 6*C*, *bottom panel*). To further explore complex formation, we performed native mass spectrometry of the P672-CCL8 complex with increasing amounts of CCL8 (Fig. S4). At a 1:1 and 2:1 ratios of CCL8:P672, we observed three peaks corresponding to 1:1 heterodimers. At a 3:1 ratio of CCL8:P672, the 1:1 heterodimeric complex continued to be observed along with monomeric and homodimeric CCL8 species. This confirms the 1:1 complex of P672 with CCL8 and indicates that CCL8 homodimers are disrupted by P672.

### Homology modeling of P672 in complex with CCL8

**Figure 7.**
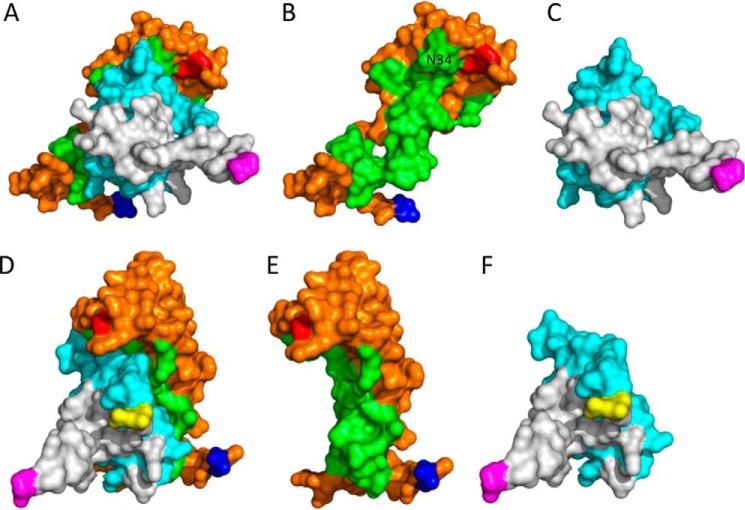
**Homology modeling of the CCL8-P672 complex.** This is a 3D structural model of P672 (*orange* and *green*) and CCL8 (*gray* and *cyan*) modeled as a 1:1 complex using the EVA1-CCL3 structure (Protein Data Bank ID 3FPU) as template and MODELLER with default parameters after alignment with the MUSCLE algorithm. The residues forming the binding surfaces predicted using Arpeggio (23) are indicated in *green* (P672) and *cyan* (CCL8). The N terminus residue of P672 (Val-1) is indicated in *blue* and the C terminus residue (Trp-104) in *red*. The N and C termini of CCL8 are indicated in *yellow* and *magenta*, respectively. *A* and *D*, P672-CCL8 complex shown in two orientations. *B* and *E*, P672-alone shown in two orientations. The position of residue Asn-34, which is in close proximity to the C-terminal Trp-104, is indicated. *C* and *F*, CCL8 alone shown in two orientations.

The published structure of the EVA1-CCL3 complex (Protein Data Bank ID 3FPU) also has 1:1 stoichiometry (16), and this template allowed the modeling of the P672-CCL8 complex using the program MODELLER (22) (Fig. 7). The model suggested that the conserved Cys residues in P672 formed disulfide bonds in a manner similar to EVA1. We next used the Arpeggio (23) Web server to identify interchain interactions and the binding surface on P672. The model suggested that key CCL8-interacting residues were present in the N terminus of P672, *i.e.* residues Glu-17—Asn-44. The model does not provide any role for the extended N terminus of P672 (*i.e.* residues Val-1—Thr-16), which do not align to EVA1 (Fig. 2*A*). Truncation of residues Val-1—Thr-16 in P672 resulted in a modest decrease in binding affinity to CCL8 (*K_d_* = 30.4 nm) suggesting that these residues may contribute to binding (Fig. S5). The model also shows that part of the binding surface (residue Asn-34) is adjacent to the C-terminal residue of P672 in the 3D structure (Fig. 7).

### Characterization of P672-EVA1 hybrid molecules

**Figure 8.**
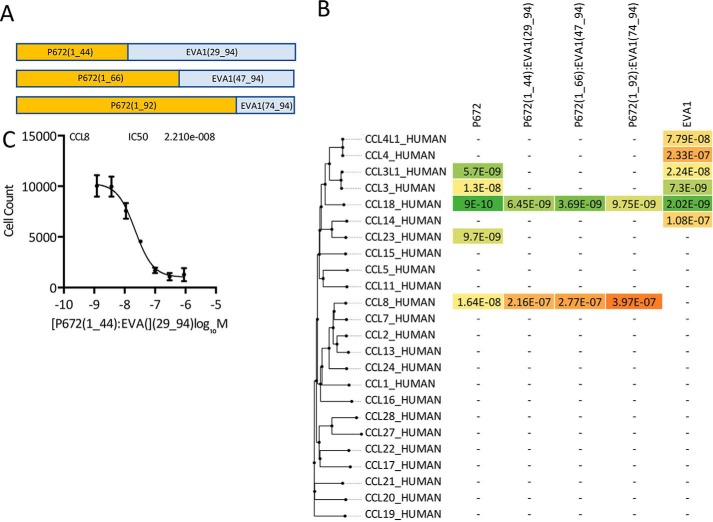
**Design and analysis of hybrid evasins.** *A*, the construction of hybrid evasins is indicated in the diagram. *B*, binding affinities (*K_d_*, m) of immobilized P672, P672_EVA1 hybrids, and EVA1 to human CC-chemokines using biolayer interferometry. High-affinity binding is indicated in shades of *green*, medium affinity in *yellow*, and low affinity in shades of *orange*. Chemokines are arranged by sequence similarity–based phylogeny. A dash (—) indicates that binding was not detected at 300 nm chemokine concentration. *C*, neutralization of CCL8-induced THP-1 monocyte cell migration by P672(1_44)-EVA1(29_94). The *y* axis shows cell count migrating through to the bottom chamber in response to an EC_80_ dose of CCL8. The Data (three technical replicates and three biological replicates) are shown as mean ± S.E. The *x* axis shows P672(1_44)-EVA1(29_94) concentration (log_10_ molar). IC_50_ was estimated by fitting an agonist response curve with four parameters as described (14).

The homology model constructed above suggested that the N-terminal residues of P672 may represent a region that confers binding to CCL8. To evaluate this hypothesis, we took advantage of the fact the EVA1 does not bind CCL8. The alignment of EVA1 with P672 (Fig. 2*A*) indicated that the predicted CCL8-binding residues are proximal to the second conserved Cys in P672. We replaced the N-terminal 29 residues of EVA1 with the equivalent N-terminal 44 residues of P672. This construct maximized the predicted CCL8 binding interface. We also created other, more extended hybrid molecules, replacing longer regions of EVA1 with the equivalent residues of P672 (Fig. 8*A*). These proteins were tagged at the C terminus with a StrepII-His tag so that changes (*e.g.* deletions) at the N terminus of P672 could be made without creating steric effects. We also constructed an identically tagged version of EVA1. We expressed these proteins as described above (Fig. S6) and characterized them for binding to target chemokines by biolayer interferometry. We initially assessed all CKBPs for binding at 300 nm chemokine concentration and then performed titration experiments to determine *K_d_* (Fig. 8*B*). EVA1 bound to the closely related chemokines CCL4L1, CCL4, CCL3L, CCL3, CCL18, and CCL14. No binding was detected to other CC chemokines, consistent with previously reported results (16). Binding to CCL3, CCL3L1, and CCL23 was lost in all the hybrids. All hybrid CKBPs bound CCL8 and CCL18. We next tested for whether the P672 (1_44)-EVA1(29_94) hybrid would neutralize CCL8 function, and we found that it did so with a mean IC_50_ of 48 nm (Fig. 8*C*). As wildtype EVA1 does not bind CCL8, these results indicate that the N-terminal 44 residues of P672, which represent 42% of the P672 protein, contain a CCL8-binding region that is transportable to another evasin.

## Discussion

In this work, we have identified, using yeast surface display approach, a novel evasin-like protein, P672_RHIPU (P672) encoded in the salivary transcriptome of the tick *R. pulchellus*. In the yeast surface display experiment, we found that only a proportion of the clonal yeast population would bind CCL8. This finding is likely due, in part, to the fact that only a proportion of clonal yeast express surface display proteins (24), consistent with our previous studies (14). As we were using yeast surface display as a screening technique rather than a quantitative measure of affinity, we explored the affinity of P672 for CCL8 using purified recombinant proteins.

Alignment to other CC chemokine–binding evasins indicates that P672 is an 8-Cys CKBP, with four disulfide bonds predicted in its structure. P672 is most closely related to EVA4 with which it shares 36% identity. In particular, these are the only two evasins identified to date that end immediately following the terminal disulfide-forming Cys residue (ending on a tryptophan) and have a tyrosine residue, instead of proline, immediately following the first disulfide-forming Cys residue. Notably this proline in EVA1 forms part of the binding surface and is the residue that targets the conserved disulfide bond in the target chemokine CCL3 (18). The significance of the substitution with tyrosine in P672 is yet unclear.

Like other evasins (14, 16, 25), P672 has several predicted *N*- and *O*-glycosylation sites, which is consistent with its significantly higher observed molecular weight and the presence of several molecular-weight species on size exclusion chromatography. PNGaseF treatment reduced the molecular weight, indicating that there is significant *N*-glycosylation. We investigated P672 glycosylation using mass spectrometry and found that *N*-glycosylation was indeed present and heterogeneous. Our data indicate that deglycosylated P672 also binds CCL8, albeit with reduced affinity. The mechanism for this action is unclear. Our mass spectrometry data indicate that residue Asn-34 in P672, which is part of the predicted binding surface, is glycosylated, and upon deglycosylation is either converted to Asp or has a single HexNAc sugar moiety attached. It is possible that these changes may affect affinity, and studies are in progress to elucidate the mechanism. Although the affinity was reduced, it did not hinder our studying the stoichiometry of the P672-CCL8 complex by native mass spectrometry.

We noted that P672 was obtained only in a single surface display tag orientation in the yeast surface display screen, with the tag positioned at the N terminus. This was unusual, as in previous screens where other evasins were identified we typically obtained clones with tags in both the N- and C-terminal positions (14). This observation is consistent with the marked discrepancy in chemokine binding affinities, as measured by biolayer interferometry, between N- and C-terminally tagged versions of P672. The reduction in binding affinity was consistent with the reduction in the ability of the protein to neutralize the target chemokine in solution. An important issue that we have not addressed here is the binding of untagged P672 to chemokines. The StrepII-His tag is necessary for rapid purification and, crucially, for binding to the biolayer interferometry biosensor, allowing comparable experiments for *K_d_* determination. An exploration of the properties of untagged P672 is necessary for future work and will require the development of strategies for P672 purification and quantifying binding affinities in the absence of a tag. Another issue that we have not addressed is the possibility that untagged or tagged P672 would bind non-chemokine proteins or molecules. This is potentially important, as such binding may mediate off-target effects if used therapeutically, and investigation of such binding is necessary in future work.

Taken together these results indicate that the C-terminal tag causes steric interference in the binding of P672 to certain chemokines. The reduction in binding affinity seen with C-terminal tagging may be explained by the close proximity of residues in the chemokine-binding surface of P672 with the C-terminal residue of P672, which could lead to steric hindrance.

P672 was identified in a screen performed with the chemokine CCL8 (also known as MCP-2) and in addition binds the closely related chemokines CCL2 (MCP-1) and CCL7 (MCP-3). These three chemokines target the common receptor CCR2, and CCL7 and CCL8 are thought to be partial agonists in comparison with CCL2 (26). CCR2 is a key regulator of monocyte and macrophage migration (27), with potential roles in diverse inflammatory disease including rheumatoid arthritis and inflammatory bowel disease (1). Notably, P672 also binds to a number of other CC chemokines. Some of these, such as CCL3 and CCL3L1, have functions in monocyte recruitment, and P672 inhibits the function of these chemokines in monocyte THP-1 cell migration assays. P672 also binds chemokines such as CCL18, CCL14, CCL23, and CCL11 with <10 nm affinity. These chemokines are involved also in lymphocyte (CCL18 (28) and CCL14 (29)), monocyte (CCL14 (29) and CCL23 (30)), and eosinophil (CCL11 (31) and CCL14 (29)) migration. As several monocyte-recruiting chemokines are bound, a potential role of P672 may be to block monocyte migration in the target hosts of the tick *R. pulchellus*. Interestingly, we observed no binding of P672 to CCL5 at chemokine concentrations below 300 nm. CCL5 is a CCR1, CCR3, and CCR5 ligand (32) and is a major chemoattractant for monocytes. The most likely explanation is that ticks such as *R. pulchellus* have other evasins, such as P467_RHIPU (14), that can bind CCL5 and thus complement the action of P672.

Tick evasins appear to bind chemokines in a mutually exclusive manner, with 8-Cys evasins binding exclusively to CC chemokines and 6-Cys evasins such as EVA3 binding exclusively to CXC chemokines. Furthermore, within each class, evasins bind only subsets of CC or CXC chemokines. This has been most clearly revealed by initial work showing that EVA4 binds to >20 CC chemokines, whereas EVA1 binds to only four CC chemokines (13). P672, like EVA1, binds only to CC chemokines, with some degree of overlap. For instance, both P672 and EVA1 are able to bind to CCL3, CCL3L1, and CCL18. EVA1 binds to CCL4, which is not bound by P672. Conversely P672 binds CCL8, whereas EVA1 does not.

An interesting observation is that the IC_50_ of P672 for inhibiting CCL8-induced cell migration is close to the *K_d_* of the CCL8-P672 interaction. In this context, CCL8 binds as an agonist to the chemokine receptors CCR1, CCR2, CCR3, and CCR5 (32), and these receptors are known to act synergistically to activate cell migration (33). Based on this understanding, one explanation for our observation is that titration of CCL8 by P672 may ablate the synergistic effects that occur at a cellular level following binding of CCL8 to its receptors. This could have larger effects on cell migration than are predicted by the *K_d_*. An alternative explanation is that affinity measured by biolayer interferometry may be underestimated, as it involves immobilization of the evasin, and this does not happen in the cell migration assay.

To begin to explore how binding specificity in the context of interactions between evasins and chemokines is created, we used native mass spectrometry. These studies showed that the stoichiometry of the P672-CCL8 complex is 1:1, the same as the previously reported EVA1-CCL3 structure. A homology model of the P672-CCL8 complex based on the EVA1-CCL3 structure and a deletion study suggested that the major CCL8-binding region is located in the N-terminal 44 residues of P672. Transferring this region, which represents 42% of the P672 protein, to EVA1 transported the CCL8 binding activity, confirming that the determinants of CCL8 binding do indeed reside in the N terminus. The reduced affinity of CCL8 binding by the hybrids suggests that either other residues outside the transported regions are needed for optimal binding to CCL8 or that the folding of the transported residues is not optimal in the context of the hybrid evasin. The loss of binding to other chemokines (CCL3, CCL3L1, and CCL23) observed in the hybrids also suggests that either other residues in P672 are needed for binding to these chemokines or that the folding of the transported residues is not optimal. The region identified as binding CCL8 represents ∼42% of P672; further experiments, beyond the scope of this work, are in progress to narrow down the binding region further. Taken together, these results suggest that regions within a tick CKBP that act to confer chemokine binding can be identified by creating hybrid evasins.

In conclusion, the identification of several novel evasins that bind distinctive subsets of CC chemokines provides the potential ability to match the chemokine-binding properties of an evasin to the disease expression patterns of a chemokine, thus generating biological tool compounds that target only disease-relevant chemokines. A key challenge is to identify functional regions within an evasin that could be used to engineer novel CKBPs with altered properties, including increased binding to certain chemokines and reduced binding to others. Analogous approaches have been used, for instance to rationally engineer antibodies with desired properties (34). In this study, by identifying and functionally characterizing a novel tick evasin, we have provided proof of concept that regions within evasins with transportable properties can be identified and manipulated to create non-natural hybrid proteins with altered characteristics. The further development of these approaches combined with structural approaches and judicious application of steric hindrance through tag location may allow us to systematically engineer novel evasins with the desired chemokine binding activities.

## Experimental procedures

### Yeast surface display

Yeast surface display screening was performed as described previously (14). Briefly, EBY100 yeast transformed with putative evasin library plasmids were induced with galactose to drive expression of the surface-displayed protein, labeled with biotinylated CCL8 (Almac) and streptavidin–Alexa Fluor 647, and then sorted using FACS; cells non-specifically binding streptavidin-AF647 were excluded using a sorting gate (Fig. 1). Then sorted cells were regrown, and a second round of sorting was performed following which cells were plated at low dilution to recover individual clones. Individual yeast clones were retested by FACS to confirm CCL8 binding. Inserts from plasmids isolated from the individual clones were then PCR-amplified and sequenced to identify the cloned evasin.

### Plasmids

The P672 N-terminal and C-terminally tagged mammalian expression plasmids were constructed in plasmid pHLSec (35) using PCR and infusion cloning as described previously (14). The purification tags were either an N-terminal His_8_-StrepII protein purification tag (HHHHHHHHSAWSHPQFEKGGGGS) or a C-terminal StrepII-His_8_ protein purification tag (TGGGGGSGGGGSGGASAWSHPQFEKLEHHHHHHHH). Following signal peptide cleavage, an additional ETG sequence predicted by SignalP 3.0 (36) was expected at the N terminus of each peptide. P672-EVA1 hybrids and EVA1 expression plasmids were made by PCR and infusion cloning into pHLSec with the C-terminal purification tag. The EVA1 plasmid used here was based on GenBank sequence EZ406190.1, which encodes EVA1 but with the variation K92N. The CCL8 expression plasmid was constructed by PCR amplification of a SUMO-CCL8 cassette and ligation-independent cloning into the vector pNIC-BIO3 (a gift from Nicola Burgess-Brown (37)) to create a HIS_6_-SUMO-CCL8 expression vector with a GSKGGYGLNDIFEAQKIEWHE C-terminal tag.

### Protein sequence analysis

We used MUSCLE in Megalign Pro (DNAStar, version 12.3.1, DNAStar Inc.) to construct the CKBP and chemokine sequence alignments and generate sequence similarity–based phylogenetic trees. These were exported to FigTree (version 1.4.2, http://tree.bio.ed.ac.uk/software/figtree/).^7^ The amino acid conservation was plotted using the EMBOSS (38) program, Plotcon (http://www.bioinformatics.nl/emboss-explorer/).^7^ Glycosylation sites were predicted using NetNGlyc1.0 (http://www.cbs.dtu.dk/services/NetNGlyc/) and NetOGlyc4.0 (http://www.cbs.dtu.dk/services/NetOGlyc/) (39).^7^ The molecular weight and pI of proteins were calculated at ExPASy (http://web.expasy.org/compute_pi/).^7^ We performed homology modeling using MODELLER (22) within PyMod 2.0 (40) with the EVA1-CCL3 structure 3FPU (16) as template. Disulfide bonds prediction was performed using the Protein Interaction Calculator (41) and interchain interactions using the Arpeggio server (23).

### CKBP production

P672 in N- and C-terminally tagged versions were produced as described previously (14). Briefly, culture supernatants from transiently transfected HEK293F cells were purified using nickel-charged IMAC Sepharose 6 Fast Flow resin (GE Healthcare) followed by size exclusion chromatography. Fractions showing absorption at 280 nm were analyzed by electrophoresis and pooled for subsequent experiments.

### Production of deglycosylated P672

The glycosylated protein precursor was prepared as described above except that upon transfection of the cells kifunensine was added to a final concentration of 7.5 μg/ml. Purified glycosylated protein was concentrated, and the solution was diluted 1:1 with 40 mm HEPES, 300 mm NaCl, and 1 mm TCEP. PNGaseF (New England Biolabs) and Endo F1 (a gift from C. Siebold, Division of Structural Biology (STRUBI), University of Oxford) was added to 2 mg of glycosylated P672 at a ratio of 1:1000 and incubated overnight at 37 °C. Degylcosylated protein was then separated from glycosylase enzymes by size exclusion chromatography into 20 mm HEPES, 150 mm NaCl, and 0.5 mm TCEP. We analyzed fractions showing absorption at 280 nm by electrophoresis on a 12% SDS-PAGE, which was stained with colloidal Coomassie.

### CCL8 production

We expressed human CCL8 (residues Gln-24 to Pro-99) in *Escherichia coli* RosettaGami^TM^ 2 (DE3) cells (Novagen). Cells were grown at 37 °C overnight in LB medium containing chloramphenicol (25 μg/ml), tetracycline (10 μg/ml), and kanamycin (50 μg/ml). This culture was then diluted 1 in 30 into Terrific Broth containing kanamycin (50 μg/ml) and grown at 37 °C until an *A*_600_ of 0.8 was reached, at which point isopropyl 1-thio-β-d-galactopyranoside was added to a final concentration of 1 mm and the culture was incubated overnight at 18 °C. Cells were pelleted by centrifugation (4000 × *g*), and the resulting pellet was frozen and stored at −80 °C. A 10-g pellet was suspended in sonication buffer (20 mm NaH_2_PO_4_, 500 mm NaCl, and 1 mm phenylmethylsulfonyl fluoride, pH 7.4) and sonicated on ice to lyse the cells. The soluble and insoluble fractions were separated by centrifugation (31,000 × *g*), and the soluble fraction was loaded by gravity flow through nickel-charged IMAC Sepharose 6 Fast Flow resin (GE Healthcare), equilibrated with binding buffer (20 mm NaH_2_PO_4_, 500 mm NaCl, pH 7.4), washed after binding with wash buffer (20 mm NaH_2_PO_4_, 1 m NaCl, 20 mm imidazole, pH 7.4), and eluted in a 4-ml elution buffer (20 mm NaH_2_PO_4_, 500 mm NaCl, 500 mm imidazole, pH 7.4) dropwise into a 20-ml cation exchange buffer (20 mm NaH_2_PO_4_, 100 mm NaCl, pH 7.4). SUMO protease (Ubl-specific protease from *Saccharomyces cerevisiae*, a gift from Dr. Ritika Sethi) was added (final concentration, 50 μg/ml) to the diluted elution and left overnight at 30 °C. The cleaved protein mixture was loaded onto a HiTrap Capto S 1-ml cation exchange column (GE Healthcare) pre-equilibrated in cation exchange buffer (20 mm NaH_2_PO_4_, 100 mm NaCl, pH 7.4) using an AKTA start. The column was then washed with 5% elution buffer (20 mm NaH_2_PO_4_, 1 m NaCl, pH 7.4) and the protein eluted with a step elution of 50% elution buffer in 1-ml fractions. The cleaved chemokine was purified to homogeneity using size exclusion chromatography (Sephadex 16/600, GE Healthcare) pre-equilibrated in a final buffer consisting of 20 mm HEPES, 500 mm NaCl, pH 7.4.

### Biolayer interferometry

Biolayer interferometry was performed on an OctetRed® system as described previously (14). Briefly, we performed cross-binding against a panel of human chemokines at 300 nm chemokine concentration. The excluded chemokines were CCL25, CCL26, and CXCL16, which non-specifically bound to the sensor, and CXCL17, CXCL4L1, and XCL2, which were not available. We evaluated binding kinetics with chemokine concentrations ranging from 300 to 0.4 nm, using a non-interacting reference protein to allow for nonspecific binding to the sensor. We used ForteBio Data Analysis 9 software to process the data and calculate association (*k*_on_), dissociation (*k*_off_), and affinity (*K_d_*) constants. Data with poor curve fits (*R*^2^ < 0.9) were excluded. The dissociation half-life or target residence time was calculated as described (14, 42) from biolayer interferometry off-rates (*k*_off_, s^−1^, as *t*_½_ = 0.693/(*k*_off_ × 60)).

### THP-1 cell migration assays

THP-1 monocyte migration assays were performed as described previously (14). Briefly, EC_80_ for each chemokine was determined using a 96-Transwell migration plate (5 μm pore size, Corning), with chemokine doses (0–100 nm) in the bottom chamber and THP-1 cells in the top chamber. Cells were counted on an ATTUNE flow cytometer, and data were analyzed in GraphPad Prism fitting an agonist response curve with four parameters. IC_50_ was determined using the above system. An EC_80_ dose of the chemokine and 0–300 nm concentration doses of the evasin were added to the bottom chamber and incubated for 30 min at 37 °C before beginning the assay. IC_50_ was calculated in GraphPad Prism fitting an inhibitor response curve with four parameters.

### Determination of glycosylation sites

Aliquots of deglycosylated P672 were reduced with dithiothreitol, alkylated using iodoacetamide, and digested with trypsin or neutrophil elastase. Peptide aliquots from tryptic digests (*n* = 3) were analyzed by LC-MS/MS using an Easy-nLC 1000 coupled to an Orbitrap LTQ-XL (Thermo Fisher Scientific). Peptides were desalted and concentrated by a C18 PepMap 100 trap (300 μm × 5 mm, 5 μm particle size, 100 Å) for 5 min at a flow rate of 20 μl/min and separated by a C18 Acclaim PepMap RSLC column (75 μm × 250 mm, 2 μm particle size, 100 Å) using a linear gradient from 0 to 64% acetonitrile:H_2_O with 0.1% formic acid during 25 min at a flow rate of 300 nl/min. For samples run in the Orbitrap-LTQ-XL, precursor ions were acquired at a mass resolution of 60,000 (*m*/*z* 400), and product ions were acquired in the ion trap. The five most intense precursor ions of charge ≥2 were selected for fragmentation by collision-induced dissociation (35 V), and dynamic exclusion settings allowed a precursor to be selected up to three times in a period of 30 s prior to being excluded for 60 s. The peptide aliquot (*n* = 1) from an elastase digest was analyzed using a Q Exactive mass spectrometer coupled with a Dionex Ultimate 3000 UPLC as described previously (43). In brief, samples were desalted online (PepMap C18, 300 μm × 5 mm, 5 μm particle size) for 1 min at a flow rate of 20 μl/min and separated on an Easy-nLC column (PepMap C18, 75 μm × 500 mm, 2 μm particle size) over 60 min using a gradient of 2–35% acetonitrile in 5% DMSO, 0.1% formic acid at a flow rate of 250 nl/min. Survey scans were acquired at a resolution of 70,000 (*m*/*z* 200); the 15 most abundant precursors of charge between 2 and 7 were selected for HCD fragmentation (35 V), and dynamic exclusion parameters were set to select a precursor ion once prior to being excluded for 60 s. The mass spectrometry proteomics data have been deposited to the ProteomeXchange Consortium via the PRIDE (44) partner repository with the dataset identifiers PXD008025 and 10.6019/PXD008025.

### Native mass spectrometry

All samples were analyzed under native conditions using a modified Q Exactive mass spectrometer (Thermo Fisher Scientific) for high-mass range measurements. CCL8 was buffer-exchanged into 200 mm ammonium acetate solution (pH 6.5), and deglycosylated P672 was buffer-exchanged into 50 mm ammonium bicarbonate buffer (pH 6.5). The proteins were initially analyzed separately using a capillary voltage of 1.2 KV and a source temperature of 100 °C. In the case of deglycosylated P672, 50 V of source-induced dissociation (SID) was added to improve desolvation. The CCL8 protein sequence was characterized from its monomers by increasing the source temperature to 200 °C and adding 30 V of SID. The two major precursor ions of CCL8 monomer, *m*/*z* 2257 and *m*/*z* 2821, were selected with an isolation window of 5 *m*/*z* and fragmented using several acceleration voltages (80, 100, 150, and/or 180 V) of HCD. All precursor and fragment ions were acquired using a mass resolution of 60,000 with an average of 3 microscans and a signal-to-noise threshold of 3. The Xtract algorithm from Xcalibur 3.0 software (Thermo Fisher Scientific) was used to determine the decharged masses of the observed ions, which were then inserted into ProSight Lite (http://prosightlite.northwestern.edu)^7^ for identification of putative post-translational modifications within a precursor and product ion tolerance of 10 ppm. Aliquots of CCL8 and deglycosylated P672 were mixed in a 1:1 ratio to reach a final concentration of 2.5 μm and incubated for 1 h at 4 °C. The mixture was analyzed using the following parameters: capillary voltage of 1.2 KV, source temperature of 130 °C, and 60 V of SID. The most intense charge state was isolated with a 100 *m*/*z* isolation window and dissociated using 100 V of HCD. In this case, precursor and dissociated products were acquired using a mass resolution of 17,500 and 60,000, respectively. Moreover, 2:1 and 3:1 ratios were also evaluated. All measurements were done in triplicate.

### Statistical analyses

GraphPad Prism was used to calculate summary statistics and tests of significance. The *p* value (probability of a type I error) reported was adjusted for multiple comparisons. The threshold (α) for a type I error was *p* < 0.05.

## Author contributions

J. R. O. E., Y. A., K. S., G. D., and S. B. conceptualization; J. R. O. E., Y. A., K. S., L. G., B. K., A. K., and S. B. data curation; J. R. O. E., Y. A., G. D., L. G., B. K., A. K., and S. B. formal analysis; J. R. O. E., Y. A., K. S., G. D., L. G., B. K., C. V. R., A. K., and S. B. investigation; J. R. O. E., Y. A., K. S., G. D., L. G., B. K., C. V. R., A. K., and S. B. methodology; J. R. O. E., Y. A., K. S., G. D., L. G., B. K., C. V. R., and A. K. writing-review and editing; K. S., G. D., L. G., B. K., C. V. R., A. K., and S. B. supervision; B. K., C. V. R., A. K., and S. B. resources; B. K., C. V. R., A. K., and S. B. software; C. V. R. and S. B. funding acquisition; A. K. and S. B. project administration; S. B. writing-original draft.

## Supplementary Material

Supporting Information

## Footnotes

7 Please note that the JBC is not responsible for the long-term archiving and maintenance of these sites or any other third party hosted site.

## Footnotes

6 The abbreviations used are:

CRS: chemokine recognition site
CKBP: chemokine-binding protein
EVA: evasin
MS/MS: tandem mass spectrometry
HCD: higher-energy collision dissociation
AF647: Alexa Fluor 647
IMAC: immobilized metal ion affinity chromatography
TCEP: tris(2-carboxyethyl)phosphine
EC: effective concentration
SID: source-induced dissociation.
