# Supplementary material for: The N-terminal domain of a tick evasin is critical for chemokine binding and neutralization and confers specific binding activity to other evasins
Source: J Biol Chem. 2018 Feb 27;293(16):6134–46. doi: 10.1074/jbc.RA117.000487 (PMC5912465; doi:10.1074/jbc.RA117.000487)
Supplement: Supporting Information [file supp_293_16_6134__index.html]

The N-terminal domain of a tick evasin is critical for chemokine binding and neutralization and confers specific binding activity to other evasins — Hybrid evasins — The N-terminal domain of a tick evasin is critical for chemokine binding and neutralization and confers specific binding activity to other evasins — Hybrid evasins — Supporting Information 

# The N-terminal domain of a tick evasin is critical for chemokine binding and neutralization and confers specific binding activity to other evasins

## Supporting Information

- Table S1 - Mass spectrometry Data
- Supplementary material Figures - Figures S1-S6
