# Supplementary material for: The N-terminal domain of a tick evasin is critical for chemokine binding and neutralization and confers specific binding activity to other evasins
Source: J Biol Chem. 2018 Feb 27;293(16):6134–46. doi: 10.1074/jbc.RA117.000487 (PMC5912465; doi:10.1074/jbc.RA117.000487)
Supplement: Supporting Information [file supp_RA117.000487_133220_3_supp_80895_p4k08r.pdf]

**Supplementary material:**

# Functional characterization of a novel tick CC chemokine binding protein identifies a transportable CCL8 binding domain

James R.O. Eaton[1,2], Yara Alenazi[1], Kamayani Singh[1], Graham Davies[1], Lucia Geis-Asteggiante[2], Benedikt Kessler[3], Carol V. Robinson[2], Akane Kawamura[1,2] & Shoumo Bhattacharya[1*]

**Contents:**

**Table S1** – P672 sequence confirmation and glycosylation sites determination (added in a separate excel file).
**Fig. S1** – Characterisation of partially deglycosylated P672
**Fig. S2** – CCL8 top-down MS/MS HCD fragmentation spectra and annotated sequence.
**Fig. S3** – Native MS decharged monoisotopic spectra of partially deglycosylated P672.
**Fig. S4** – Native MS of CCL8:P672 complex with increasing amounts of CCL8
**Fig. S5** – Expression and characterization of P672(17_104).
**Fig. S6** – Expression and purification of evasin hybrid molecules.

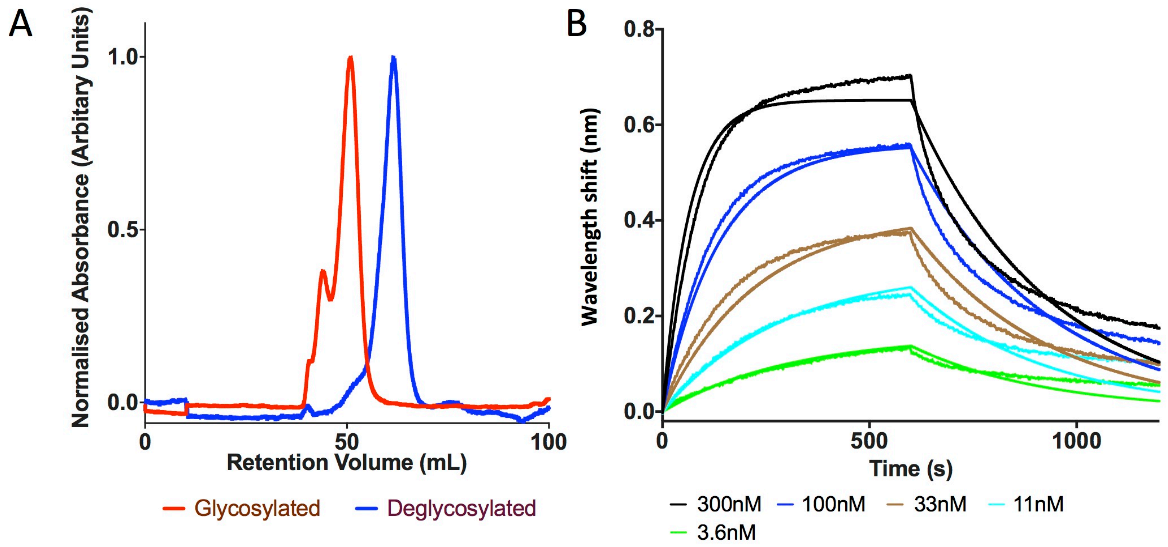


**Fig. S1 – Characterisation of partially deglycosylated N-terminally tagged P672. A**. Size exclusion chromatography traces of glycosylated P672 (red) and partially deglycosylated P672 (blue) using PNGaseF and EndoF1. **B.** Biolayer interferometry sensorgram of P672 (partially deglycosylated) binding to CCL8. Plots display wavelength shift (Y-axis, nm) versus time (X-axis, seconds). Solid lines indicate collected data, dashed lines indicate fitted data. $K_d$ (M) = 7.25E-08, target residence time = 3.8 minutes.

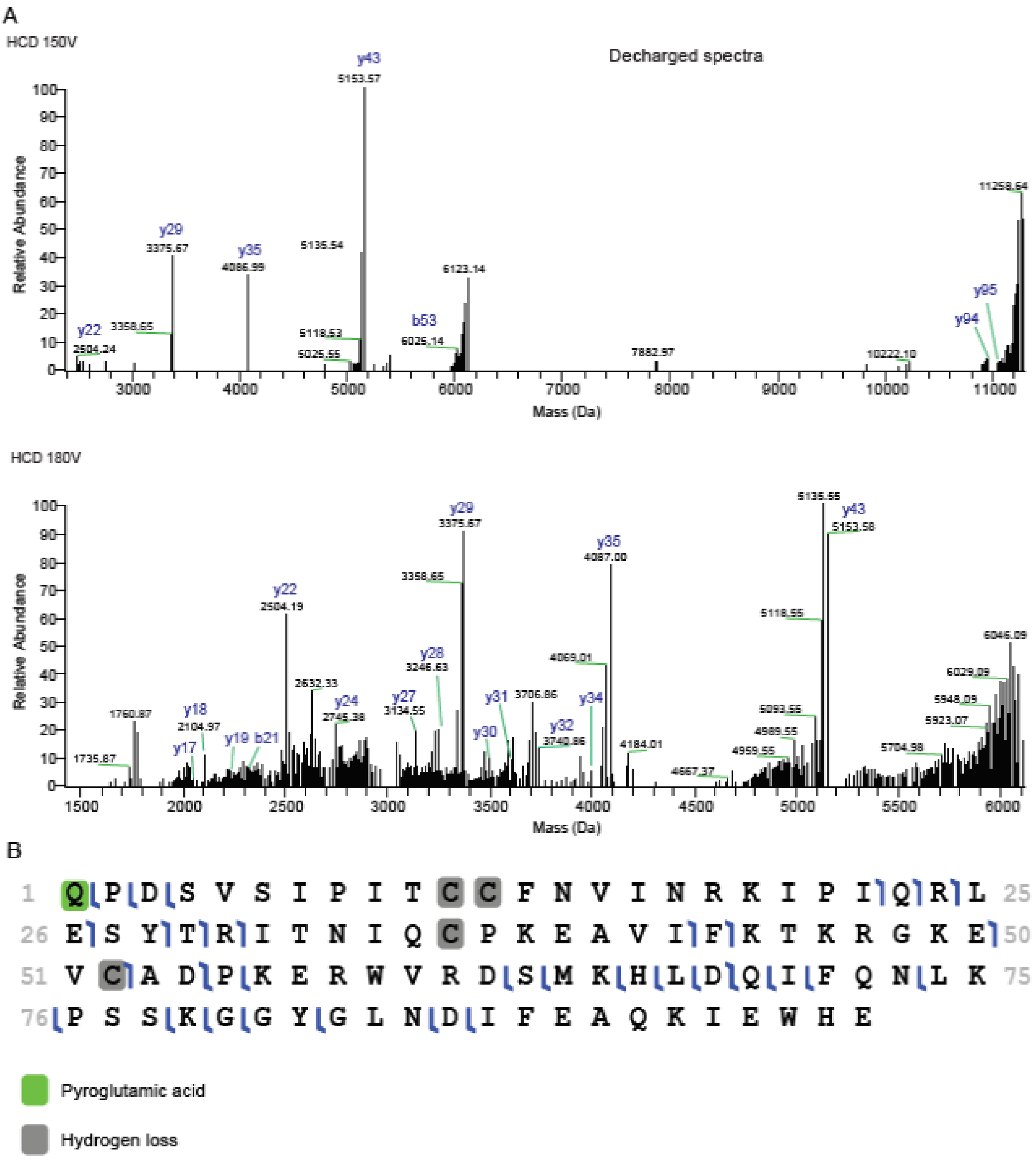


**Fig. S2 - CCL8 top-down MS/MS HCD fragmentation spectra and annotated sequence.** CCL8 monomer was characterized by selecting precursor ions (isolation window of 5 m/z) and fragmenting them using higher collision induced dissociation (HCD). **A.** Decharged monoisotopic spectra obtained for *m/z* 2821 (z=5) when fragmented with 150 and 180 V. **B.** Annotated sequence fragmentation pattern showing a 33% residue cleavage. The protein sequence included the presence of 2 disulfide bonds and the formation of pyroglutamic acid at the N-terminal glutamine during ionization (monoisotopic mass of 11276.76 Da).

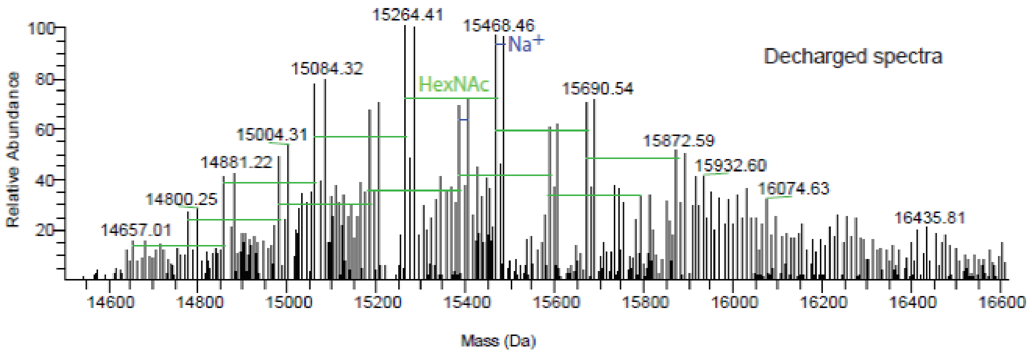


**Fig. S3 - Native MS decharged monoisotopic spectra of partially deglycosylated P672.** The decharged spectra shows monoisotopic masses of several P672-RHIPU modified forms. Mass differences suggesting the presence of sodium adducts and 5 to 6 HexNAc mass additions (+203 Da) were observed. The presence of partial glycosylations was supported by shotgun proteomics analyses.

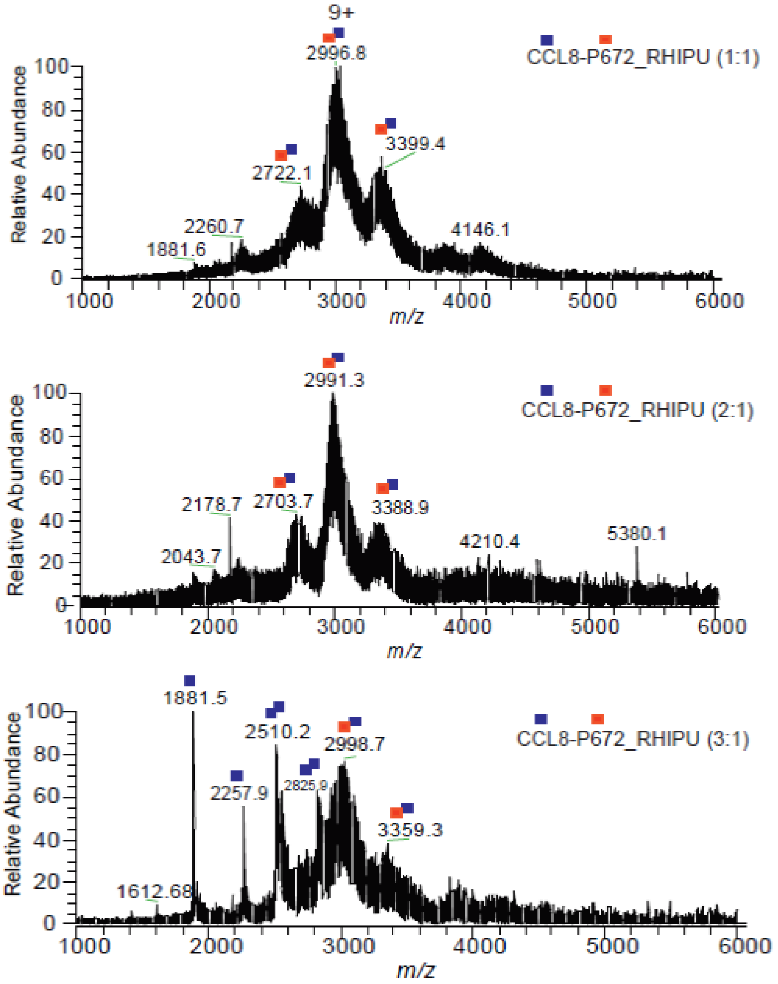


**Fig. S4 - Native MS of CCL8:P672 complex with increasing amounts of CCL8**

The P672:CCL8 complex was formed at ratios 1:1, 2:1 and 3:1 as indicated in top, middle and bottom panels. At 1:1 and 2:1 ratios the CCL8:P672 heterodimer is the prominent component observed and, as the ratio increase to 3:1, CCL8 monomer and homodimer are also observed.

Peaks are indicated as CCL8 (blue squares) or P672 (red squares). Monomers are indicated as a single square, and homo or heterodimers as double squares. Y-axis indicates relative abundance, X-axis indicates *m/z* (mass/charge) ratio.

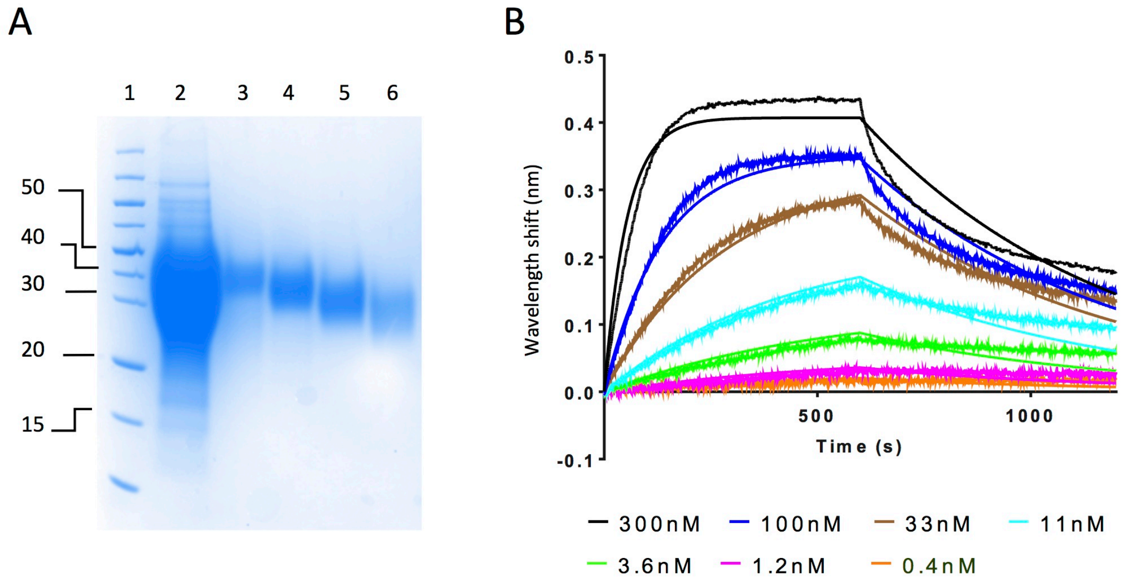


**Fig. S5 - Expression and characterization of P672(17_104)**

**A.** Expression and purification of C-terminally tagged P672(17_104). SDS-PAGE gel showing molecular weight marker (lane 1), Ni-NTA captured protein (lane 2) and size exclusion chromatography fractions (lanes 3-6) of C-terminally tagged P672(17-104) stained with colloidal Coomassie blue.

**B.** Biolayer interferometry sensorgram of P672(17_104) binding to CCL8. Plots display wavelength shift (Y-axis, nm) versus time (X-axis, seconds). Solid lines indicate collected data, dashed lines indicate fitted data. $K_d$ (M) = 3.04E-08, target residence time = 6.7 minutes.

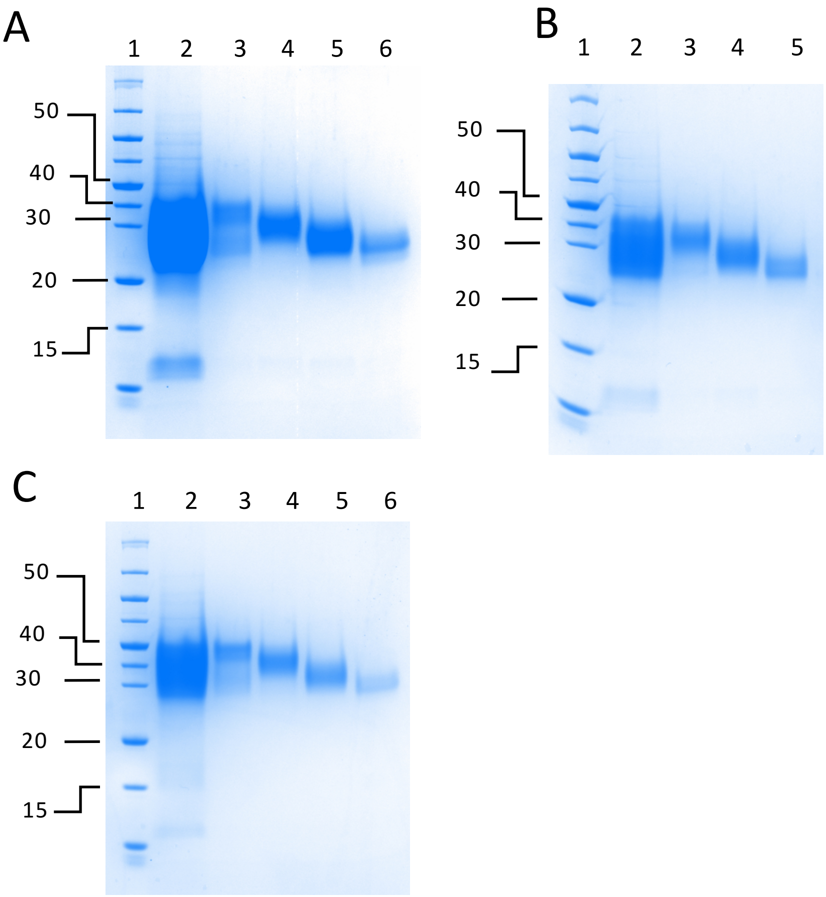


**Fig. S6 - Expression and purification of hybrid evasin proteins. A.** SDS-PAGE gel of molecular weight marker (lane 1), Ni-NTA captured protein (lane 2) and size exclusion chromatography fractions (lanes 3-6) of P672(1_44): EVA1(29_94). **B.** SDS-PAGE gel of molecular weight marker (lane 1), Ni-NTA captured protein (lane 2) and size exclusion chromatography fractions (lanes 3-5) of P672(1_66): EVA1(47_94). **C.** SDS-PAGE gel of molecular weight marker (lane 1), Ni-NTA captured protein (lane 2) and size exclusion chromatography fractions (lanes 3-6) of P672(1_92): EVA1(74_94).
